# Perilipin 2 (PLIN2)-Deficiency Does Not Increase Cholesterol-Induced Toxicity in Macrophages

**DOI:** 10.1371/journal.pone.0033063

**Published:** 2012-03-12

**Authors:** Se-Hee Son, Young-Hwa Goo, Benny H. Chang, Antoni Paul

**Affiliations:** 1 Center for Cardiovascular Sciences, Albany Medical College, Albany, New York, United States of America; 2 Department of Molecular and Cellular Biology, Baylor College of Medicine, Houston, Texas, United States of America; University of Florida, United States of America

## Abstract

Interventions on macrophages/foam cells to redirect intracellular cholesterol towards efflux pathways could become a very valuable addition to our therapeutic arsenal against atherosclerosis. However, certain manipulations of the cholesteryl ester cycle, such as the inhibition of ACAT1, an ER-resident enzyme that re-esterifies cholesterol, are not well tolerated. Previously we showed that targeting perilipin-2 (PLIN2), a major lipid droplet (LD)-associated protein in macrophages, prevents foam cell formation and protects against atherosclerosis. Here we have assessed the tolerance of PLIN2-deficient bone marrow derived macrophages (BMM) to several lipid loading conditions similar to the found during atherosclerosis development, including exposure to modified low-density lipoprotein (mLDL) and 7-ketocholesterol (7-KC), a free cholesterol (FC) metabolite, in media with or without cholesterol acceptors. BMM isolated from mice that do or do not express PLIN2 were tested for apoptosis (TUNEL and cleaved caspase-3), ER stress (CHOP induction and XBP-1 splicing), and inflammation (TNF-α and IL-6 mRNA levels). Like in other cell types, PLIN2 deficiency impairs LD buildup in BMM. However, while most stress parameters were elevated in macrophages under ACAT inhibition and 7-KC loading, PLIN2 inactivation was well tolerated. The data support the safety of targeting PLIN2 to prevent foam cell formation and atherosclerosis.

## Introduction

Lipid-laden macrophages or foam cells are fundamental to the formation and progression of atherosclerosis [1]. They play the central role of removing the mLDL retained in the arterial intima and, consequently, need to deal with a large influx of cholesterol. The lipoprotein-derived cholesterol ester (CE) is hydrolyzed in the lysosome and FC is released to the cytoplasm. Part of this FC can be effluxed to extracellular acceptors such as apolipoprotein A1 or high-density lipoprotein, but a large portion of it is re-esterified and stored as CE in cytoplasmic LDs [2]. The fate of intracellular cholesterol may influence the development of atherosclerosis, and while re-esterification promotes foam cell formation and cholesterol retention at the arterial wall, efflux facilitates reverse cholesterol transport. Since cholesterol is effluxed as FC, conceivably efflux can be enhanced by increasing the intracellular FC/CE ratio. However, certain interventions aiming to prevent CE accumulation as a strategy to increase efflux have actually been detrimental. For example, cholesterol is re-esterified by acetyl-CoA acetyltransferase1 (ACAT1) [3] and, therefore, ACAT1 inhibition would be expected to protect against atherosclerosis. Conversely, most evidence supports that decreasing ACAT1 activity actually promotes atherosclerosis, which has been attributed to side effects caused by excess of FC trafficking to the ER membrane, including ER stress, increased synthesis of proinflammatory cytokines, and macrophage death [4]–[7]. Hence, the foam cell's tolerance to other manipulations of the cholesteryl ester cycle needs to be carefully evaluated.

LDs consist of a core of neutral lipid, mainly CE and triglycerides, coated by a monolayer of phospholipids, FC and proteins [8]. The most important structural proteins associated to LDs are the members of the PAT family, named after **P**erilipin, **A**dipose differentiation-related protein and **T**ail-interacting protein of 47 kDa [9]. In macrophages the main PAT protein is PLIN2 (also known as ADFP, ADRP or adipophilin before an unifying nomenclature was adopted) [10]. PLIN2 plays an important role in lipid trafficking in macrophages. PLIN2 expression increases following lipid loading in several monocytic cell lines and in primary macrophages [11]–[15]. *In vivo*, PLIN2 mRNA is upregulated in carotid endarterectomy specimens and in atherosclerosis-studded arteries of apoE^−/−^ mice compared to control healthy arteries [12], [14]. PLIN2 overexpression in THP-1 macrophages increases lipid accumulation upon incubation with acetylated (ac) LDL, while PLIN2 downregulation reduces it [14]. Peritoneal macrophages isolated from PLIN2-deficient mice display less cytoplasmic LDs and decreased CE following incubation with oxidized (ox) LDL or acLDL and, consistently, foam cells in lesions of PLIN2 knockout mice contain less LDs than those of their wild-type (WT) littermates [12]. Data from cholesterol trafficking experiments support that, by facilitating CE accumulation in LDs, PLIN2 hinders cholesterol efflux [12], [14]. Previously we showed that PLIN2 deficiency protects apoE-deficient mice against atherosclerosis [12]. Here we have studied the response of BMM isolated from mice that do or do not express PLIN2, in terms of apoptosis, ER stress and inflammation, to multiple culture conditions that mimic the lipid environment in different stages of atherosclerosis development. While there was toxicity associated to ACAT1 inhibition and FC loading, the data support that PLIN2 inactivation is well tolerated by macrophages.

## Materials and Methods

### Ethics statement

All animal experiments were conducted following a protocol approved by the Institutional Animal Care and Use Committee at Albany Medical College (protocol number 901397). All efforts were made to minimize suffering.

### Cell culture and experimental design

BMM isolated from femurs of PLIN2-deficient mice in C57BL/6J background [16] and age and gender matched WT controls were cultured in RPMI-1640 media supplemented with 10% heat-inactivated fetal bovine serum (FBS) and 15% (v/v) L929 cell-conditioned medium, that served as the source of M-CSF [17]. At passage three BMM were subcultured into two six-well plates and remained untreated, or were treated for 20 hours with acLDL (50 µg/ml; Biomedical Technologies), an ACAT inhibitor (ACATi; 10 µg/ml; Sandoz 58-035, Sigma-Aldrich), the combination of acLDL plus ACATi, or 7-KC (50 µM; Sigma-Aldrich). These concentrations of acLDL combined with ACATi or 7-KC alone have previously been shown to induce ER-stress and apoptosis in macrophages [18]–[21]. Because the 7-KC was dissolved in ethanol, we also included an ethanol/vehicle group. To mimic the limited access to extracellular cholesterol acceptors in the core of advanced lesions, the study was performed in parallel in media without FBS. The study design is summarized in Table 1. Nine mice of each genotype were included, and their BMM were used to obtain protein (n = 3), RNA (n = 3) or for staining (n = 3).

**Table 1:** Experimental design.

Untreated control				acLDL (50 µg/ml)				ACATi (10 µg/ml)				acLDL+ACATi				Control (ethanol)				7-KC (50 µM)			
+FBS		−FBS		+FBS		−FBS		+FBS		−FBS		+FBS		−FBS		+FBS		−FBS		+FBS		−FBS	
**WT**	**KO**	**WT**	**KO**	**WT**	**KO**	**WT**	**KO**	**WT**	**KO**	**WT**	**KO**	**WT**	**KO**	**WT**	**KO**	**WT**	**KO**	**WT**	**KO**	**WT**	**KO**	**WT**	**KO**

### Western blots and quantitative PCRs

Fifteen µg of protein were resolved by SDS-PAGE and transferred onto a PVDF membrane. The antibodies used for Western blot included a rabbit anti-PLIN2 (Novus Biologicals), a rabbit anti-GADD153 [C/EBP-homologous protein (CHOP), Santa Cruz Biotecnologies], a rabbit anti-cleaved caspase-3 (Cell Signaling), and a rabbit anti-GAPDH (Sigma-Aldrich). RNA was isolated with TRIzol (Invitrogen), digested with DNase I and purified using the Absolutely RNA miniprep kit (Agilent Technologies). Reverse transcription of 500 ng of total RNA was performed with SuperScript III (Invitrogen). Primer sequences were designed with Primer3 software [22] and are available in Table S1. Quantitative real-time PCR (qPCR) was performed with SYBR Green QPCR Master Mix from Stratagene (Cedar Creek, TX) using 40 amplification cycles (95°C for 30 s, 55°C for 1 min, 72°C for 1 min). Relative gene expression levels were determined from threshold cycle (Ct) values normalized to cyclophilin A as we previously described [12]. The splicing of X-box binding protein 1 (XBP-1) was assessed by RT-PCR using primers that detect both unspliced (205 bp) and spliced (179 bp) isoforms. PCR products were separated in 6% polyacrylamide gels and visualized by ethidium bromide staining. Relative protein levels and XBP-1 mRNA splicing were quantified using ImageJ software.

### Oil red O and TUNEL stainings

For oil red O (ORO) staining BMM were fixed with 3.7% paraformaldehyde, washed with phosphate buffered saline (PBS) and stained for 30 minutes with ORO (Sigma-Aldrich; 0.3% w/v in 60% isopropanol). Nuclei were stained with hematoxylin (Vector Laboratories). TUNEL staining was performed with the DeadEnd Fluorometric TUNEL system (Promega), and cell nuclei were stained with DAPI (Sigma-Aldrich). Cell number and TUNEL-positive cells in six to ten randomly selected fields per sample were quantified blindly by two independent investigators.

### Data analysis

Statistical significance of the effect of the three independent variables included in this study (PLIN2 genotype, treatment, and presence or absence of FBS) was determined by three way ANOVA using the SigmaStat software. Pairwise comparisons were performed by the Holm-Sidac method. In addition, a two-tailed Student's *t*-test was used to compare WT and PLIN2-deficient macrophages within each treatment group. Differences were considered significant at p<0.05. Results are shown as mean ± SEM.

## Results

### Role of PLIN2 in LD formation in BMM

**Figure 1:**
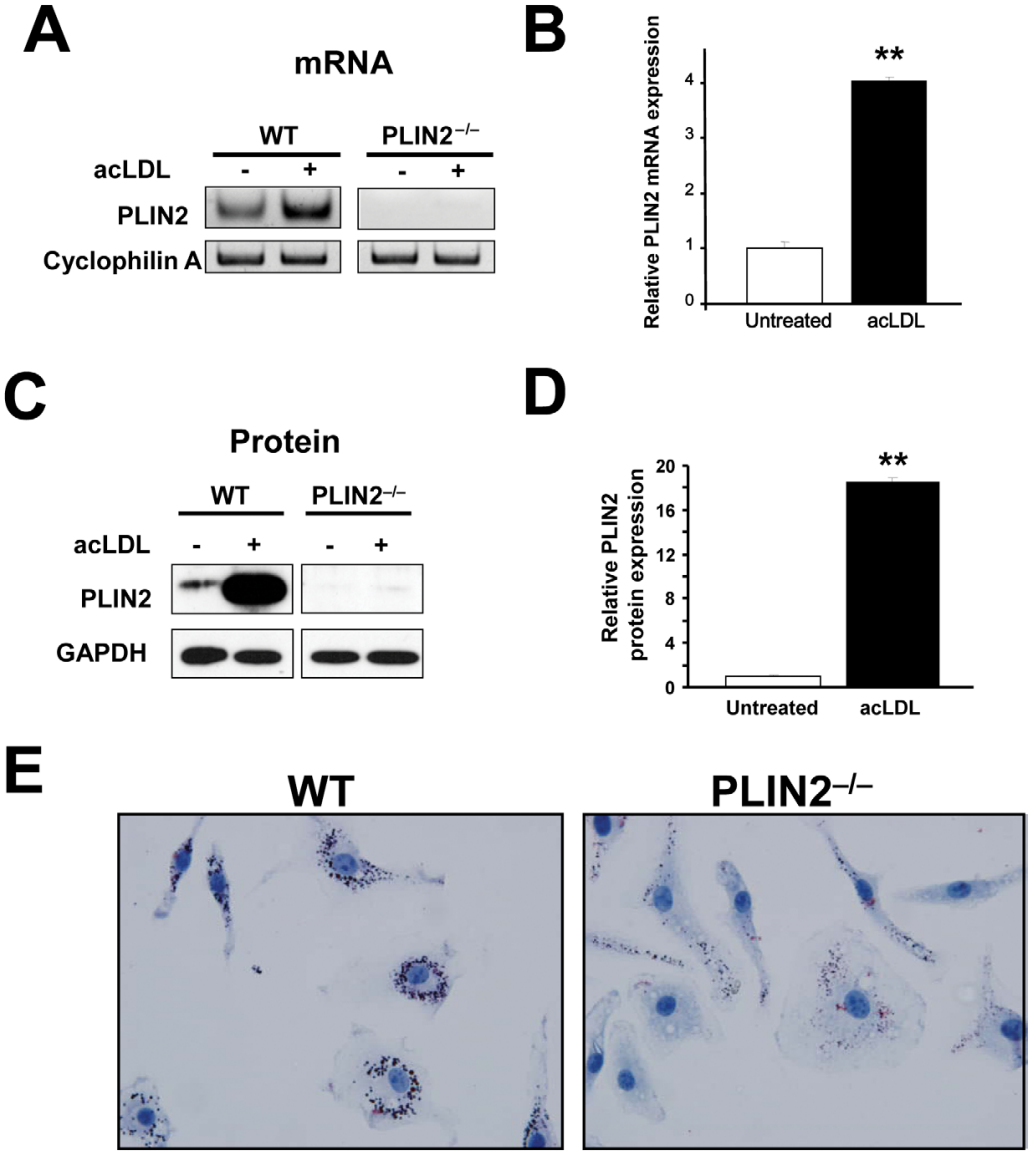
PLIN2 is upregulated by lipid loading in BMM, and PLIN2 deficiency inhibits LD accumulation. (**A**) Representative RT-PCR analysis (29 cycles) showing PLIN2 mRNA induction by acLDL (50 µg/ml for 20 h) in BMM. (**B**) qPCR analysis of PLIN2 mRNA expression relative to cyclophilin A in WT BMM macrophages. n = 3, **p<0.01. (**C**) Representative Western blot and (**D**) quantification of PLIN2 protein relative to GAPDH in BMM cultured with or without acLDL. n = 3, **p<0.01. (**E**) Oil red O staining in WT (left) and PLIN2-deficient (right) BMM cultured with acLDL.

BMM can be expanded *in vitro*, and this allowed us to obtain enough cells to simultaneously compare the response between WT and PLIN2-deficient macrophages under multiple culture conditions. Because the role of PLIN2 on LD formation in BMM had not been studied, first we asked whether this is similar to the previously described in other primary macrophages and monocyte/macrophage cell lines [12], [14]. As seen in Figure 1A and 1B, qPCR analysis showed that upon incubation with acLDL there was a significant (∼4-fold) increase in PLIN2 mRNA. At the protein level, while by immunoblot PLIN2 was barely detectable in untreated cells, it was ∼18-fold higher in acLDL-treated BMM (Figure 1C and 1D). Note that when not bound to LDs PLIN2 is rapidly degraded by the proteasome, and very low levels of the protein are expected in non-lipid laden cells [23]. On the other hand, as seen in Figure 1E, ORO staining showed abundant cytoplasmic LDs in acLDL-treated WT BMM, but the absence of PLIN2 markedly reduced LD buildup. Thus, in agreement with previous observations in multiple tissues and cell types, including macrophages, in BMM PLIN2 mRNA and protein are upregulated by lipid loading, and the absence of PLIN2 hinders LD formation.

### PLIN2 inactivation does not induce apoptosis in BMM

Cholesterol loading can induce macrophage apoptosis [7]. If high rates of apoptosis overpower the efferocytosis (clearance of apoptotic bodies) capacity of the neighboring macrophages, the accumulated apoptotic bodies may become disrupted and release their proatherogenic cargo [24]. In early atherogenesis foam cell formation is primarily due to mLDL uptake. In advanced lesions the cells are also exposed to FC and its oxidized derivates, in an environment relatively poor in cholesterol acceptors. Thus, our treatments included acLDL, a form of mLDL avidly taken-up by macrophages, and 7-KC, a FC oxidation product found in atherosclerotic lesions [25]. Media with or without FBS allowed or limited access to extracellular acceptors, respectively.

**Figure 2:**
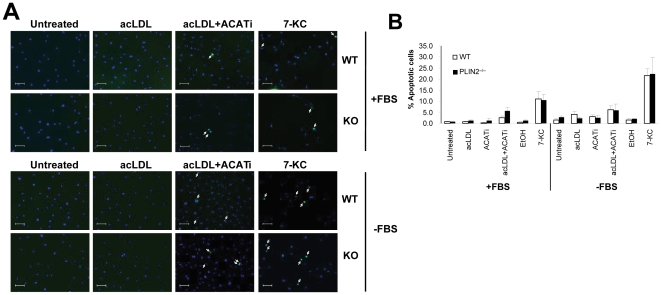
Analysis of apoptosis: TUNEL staining. (**A**) Representative images of TUNEL staining in untreated and acLDL, acLDL+ACATi and 7-KC-treated BMM isolated from WT and PLIN2-deficient mice cultured with or without FBS supplementation. TUNEL positive cells (green) are pointed by arrows. Bar = 50 µm. (**B**) Quantification of TUNEL positive cells in all the treatment groups (n = 3). The results of a three-way ANOVA analysis of these data are shown in Table S2 online.

Apoptosis was assessed by two commonly used methods: TUNEL to detect DNA fragmentation and Western blot to detect cleaved caspase-3. The TUNEL results are summarized in Figure 2. Briefly, in macrophages cultured with FBS there was a low (∼1%) rate of apoptosis in the untreated and in the ACATi and acLDL-treated groups, and was not affected by the PLIN2 genotype. Treatment with acLDL+ACATi increased the rate of apoptosis to ∼2.6% in WT macrophages and to ∼5.6% in PLIN2-deficient macrophages, but the difference between genotypes did not reach statistical significance. The effect of 7-KC was more marked, but similar between genotypes: 11.1% in PLIN2^+/+^ macrophages and 10.6% in PLIN2^−/−^ macrophages. In general, the rate of apoptosis increased when the macrophages were cultured without FBS, ranging between 1.5 and 4% in the control, acLDL and ACATi groups. Again, acLDL treatment did not induce apoptosis in PLIN2-deficient macrophages and in this case the rate of apoptosis in acLDL+ACATi-treated cells was similar in both genotypes (6.1% in PLIN2^+/+^ macrophages and 6.0% in PLIN2^−/−^ macrophages). 7-KC treatment also increased the number of TUNEL positive cells to a comparable level in both genotypes: 21.7 and 22.4% in WT and PLIN2-deficient macrophages, respectively. Three way ANOVA analyses showed that FBS depletion significantly increased apoptosis. Among the treatments, the effect of 7-KC was highly significant. While comparisons between the acLDL+ACATi treatment and each one of the other treatment groups yielded p values<0.05, these values did not reach the critical level to be considered significant in this analysis. The PLIN2 genotype did not influence the results (p = 0.650). The ANOVA results are summarized in Table S2. In addition, *t*-test analyses did not show significant differences between WT and PLIN2-deficient macrophages under any condition.

**Figure 3:**
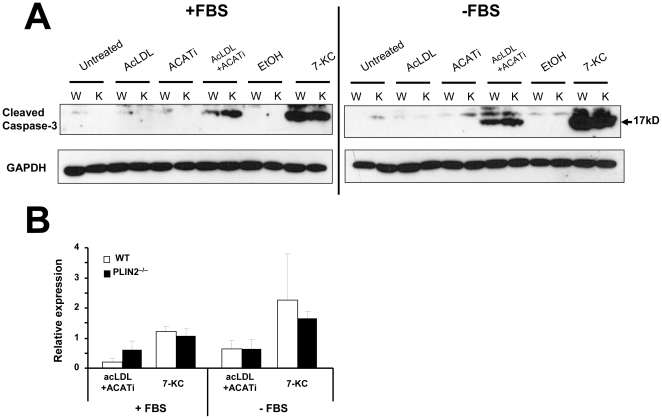
Analysis of apoptosis: caspase-3 activation. (**A**) Representative Western blots to detect the 17 kD subunit of active caspase-3. The band was detectable only in the acLDL+ACATi and 7-KC-treatment groups. (**B**) Quantification of the 17 kD caspase-3 fragment relative to GAPDH in the acLDL+ACATi and 7-KC-treatment groups (n = 3).

The levels of the 17 kD subunit of active caspase-3 paralleled the TUNEL data. As seen in Figure 3A, cleaved caspase-3 was detectable only in acLDL+ACATi and 7-KC-treated macrophages. Treatment with acLDL did not induce caspase-3 activation in PLIN2-deficient cells whether they were cultured with or without FBS. Interestingly, capase-3 activation was higher in FBS+/acLDL+ACATi-treated PLIN2^−/−^ macrophages than in their WT counterparts, although this difference was not statistically significant (Figure 3B). In summary, we only observed a modest trend of increase in apoptosis markers in PLIN2-deficient cells when they were cultured with acLDL in the presence FBS and with the ACAT activity inhibited pharmacologically. Under all other conditions, PLIN2 deficiency did not increase apoptosis.

### PLIN2 inactivation does not increase lipid-induced ER stress in BMM

**Figure 4:**
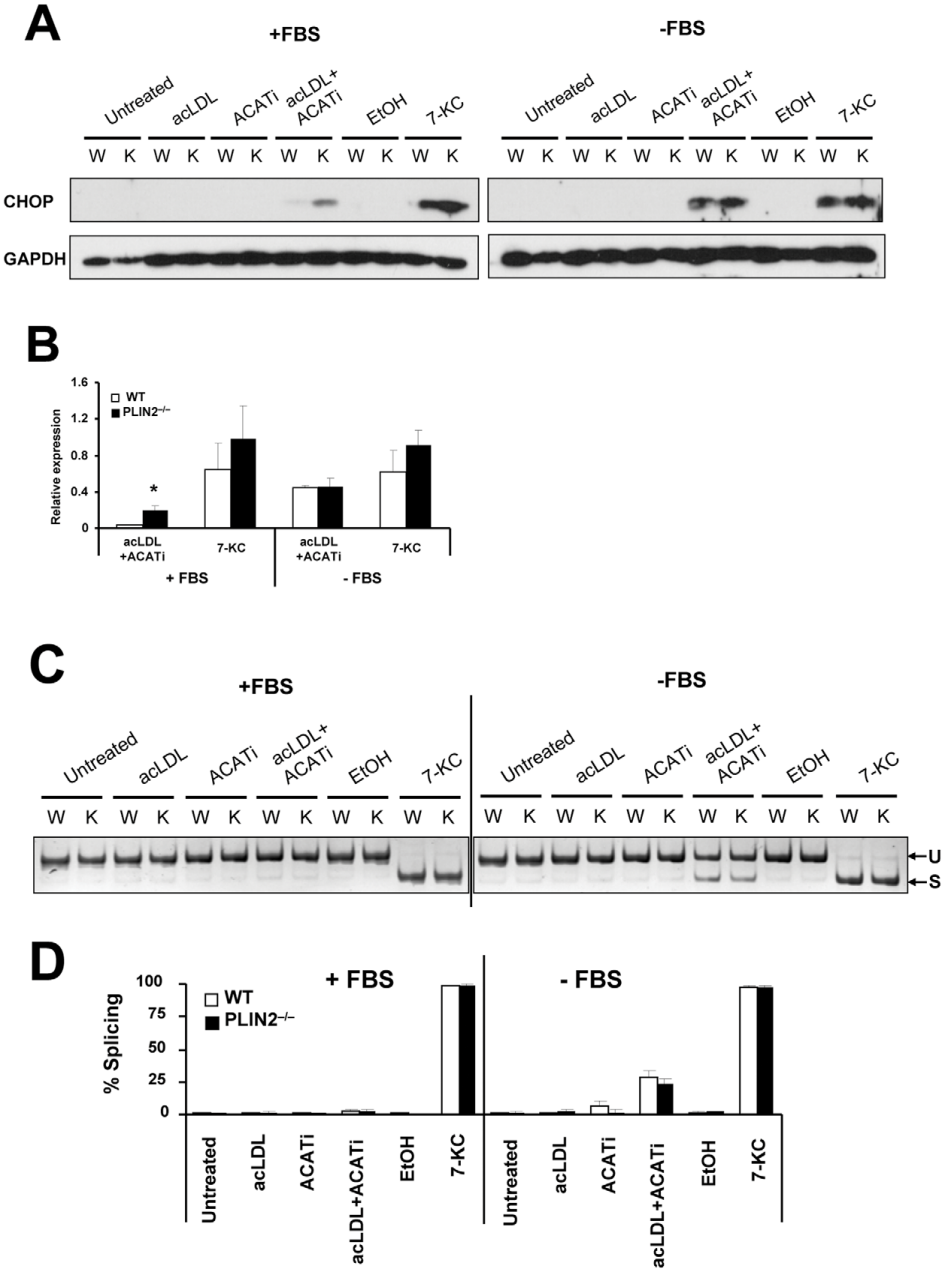
Analysis of ER stress: CHOP induction and XBP-1 splicing. (**A**) Representative Western blot showing CHOP induction only in the acLDL+ACATi and 7-KC-treatment groups. The band was detectable only in the acLDL+ACATi and 7-KC-treatment groups. (**B**) Quantification of CHOP relative to GAPDH in the acLDL+ACATi and 7-KC-treatment groups (n = 3; *p<0.05 respect to WT). (**C**) XBP-1 unspliced (U) and spliced (S) mRNAs in the different treatment groups. (**D**) Quantification of splicing. The bars represent % of spliced XBP-1 with respect to the total (spliced+unspliced) XBP-1. n = 3.

The ER is considered the main site of cholesterol-induced toxicity in foam cells [5], [26]. We asked whether PLIN2 deficiency could induce a subapoptotic level of ER stress that in a multi-hit scenario could facilitate apoptosis induction by other factors [24]. We analyzed two parameters commonly used to detect activated unfolded protein response (UPR), the adaptive response of the ER to stress: the induction of CHOP and the splicing of XBP-1 [27]. As seen in Figure 4A, CHOP, a proapoptotic transcription factor [5], [21], [28], was robustly induced by 7-KC and to a lesser extend by mLDL+ACATi, the two conditions that increased apoptosis. Treatment with acLDL or ACAT inhibitor alone did not cause CHOP induction in PLIN2-deficient BMM whether they were cultured with or without FBS, and PLIN2-deficient and WT BMM responded to 7-KC indistinguishably. Interestingly, compared to WT BMM CHOP was moderately elevated in PLIN2-deficient macrophages in the FBS+/acLDL+ACATi group (Figure 4B).

The highest rates of XBP-1splicing were also observed in 7-KC-treated macrophages cultured both with and without FBS, and in the FBS−/acLDL+ACATi group. PLIN2 inactivation did not increase XBP-1 splicing in any treatment group, including the FBS+/acLDL+ACATi group (Figure 4C and 4D). Thus, both UPR markers increased under conditions known to induce ER stress, i.e. 7-KC loading and treatment with acLDL+ACATi in the absence of FBS [29], [30]. However, PLIN2 inactivation did not alter these markers except for a moderate increase in CHOP induction in macrophages treated with acLDL+ACATi in the presence of FBS.

### Lack of PLIN2 does not increase the synthesis of TNF-α and IL-6 in BMM

**Figure 5:**
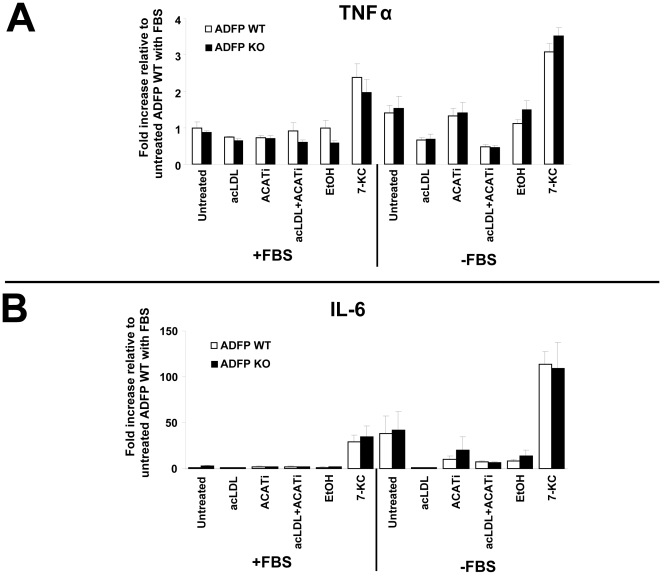
Analysis of inflammation: TNFα and IL-6 mRNA. qPCR analysis of TNF-α (**A**) and IL-6 (**B**) mRNA expression relative to cyclophilin A. (n = 3). The results of three-way ANOVA analyses of these data are shown in Tables S3 and S4 in the online data supplement.

Inflammation is a major player in atherogenesis [31]. Previous work has shown that FC accumulation in macrophages can induce the synthesis of inflammatory cytokines such as tumor necrosis factor-α (TNF-α) and interleukin-6 (IL-6) [6], [32], [33]. Thus, we analyzed, by qPCR, the relative mRNA expression of these two cytokines. As seen in Figure 5, in macrophages cultured in the presence of FBS 7-KC treatment resulted in ∼2.5 and ∼30-fold induction of TNF-α and IL-6 mRNA, respectively. In the remaining treatment groups the mRNA levels were similar to those in the control group. FBS deprivation resulted in a variable induction of both cytokines, but both transcripts were higher in 7-KC-treated BMM. No significant differences between genotypes were observed when comparisons within each individual treatment group were performed by *t*-test under any experimental condition. Three-way ANOVA analysis showed that FBS depletion and 7-KC treatment significantly increased the levels of both transcripts, while the PLIN2 genotype did not influence the results. The ANOVA results are summarized in Tables S3 and S4.

## Discussion

Arguably, proving the safety of a molecular intervention with potential therapeutic use is as relevant as proving its efficiency. Previously we showed that PLIN2 ablation does not affect acLDL binding and cholesterol uptake by macrophages, but it hampers the foam cell's ability to accumulate surplus of cholesterol as CE in cytoplasmic LDs [12]. Thus, under PLIN2 deficiency part of the cholesterol that would be stored in LDs will need to be redistributed within the cell. On one hand this may be beneficial because some of it may traffic to compartments from where it can be effluxed, and this is probably why macrophages lacking PLIN2 showed increased cholesterol efflux [12]. However, this may also cause more FC recirculation to the ER, which inherently implies more risk of ER stress. In this study we have assessed the tolerance of PLIN2-deficient macrophages to different forms of lipid exposure, including milder conditions expected in early atherogenesis such as exposure to mLDL in the presence of extracellular cholesterol acceptors, but also two more stressing conditions expected within advanced lesions, namely exposure to mLDL in the absence of extracellular acceptors and exposure to free and oxidized cholesterol derivates. Treatment with an ACAT inhibitor allowed comparing the effects of PLIN2 deficiency to those of a well-known inducer of ER stress-mediated apoptosis. Collectively, our data support that PLIN2 deficiency is well tolerated by the macrophage. Consistent with observations in macrophages from other sources, PLIN2 deficiency reduced LD formation in BMM. However, conversely to what happened under ACAT inhibition, acLDL treatment did not induce ER stress and did not increase the rate of apoptosis in PLIN2^−/−^ macrophages even when they were cultured in the absence of FBS, a condition that hampers the cell's ability to efflux cholesterol and, consequently, could increase its dependence on cholesterol storage. In addition, while 7-KC induced ER stress, apoptosis and inflammation in macrophages cultured with and without FBS, these effects were similar in macrophages of both genotypes. A possible explanation for the low toxicity associated to PLIN2 deficiency compared to ACAT inhibition may rely on the cellular localization of the proteins. The ER membrane is extraordinarily poor in cholesterol, and while FC accumulation at the ER has a marked proapoptotic effect, macrophages show better tolerance to FC accumulation at the plasma membrane [5], suggesting that the site of intervention actually matters when we target the cholesterol ester cycle. Interestingly, we observed a moderate increase in TUNEL positive cells, cleaved caspase-3 and CHOP induction in PLIN2-deficient macrophages treated simultaneously with acLDL and an ACAT inhibitor in the presence of FBS. The increase in CHOP and apoptosis markers was limited to this group, and was markedly lower than the seen after FBS withdrawal. Altogether, these data suggest that while PLIN2 deficiency may actually increase cholesterol recirculation through the ER, this does not cause ER stress unless re-esterification is blocked.

The existence of a cholesterol esterification/hydrolysis cycle in macrophages has been known for decades [2]. However, there are still many missing pieces in a puzzle that we need to solve if we want to tackle cholesterol trafficking to prevent foam cell formation and atherosclerosis. Previously we showed that targeting PLIN2 reduces foam cell formation and atherosclerosis [12]. Here we show that PLIN2 ablation is well tolerated by macrophages. Overall, the data support the therapeutic potential of interventions at the LD level aiming to mobilize intracellular cholesterol towards efflux and reverse transport pathways to slow-down atherogenesis.

## Supporting Information

Table S1 **Primer sequences.**
(DOCX)

Table S2 **Three way ANOVA analysis of the TUNEL data.**
(DOCX)

Table S3 **Three way ANOVA analysis of the TNF-α mRNA levels.**
(DOCX)

Table S4 **Three way ANOVA analysis of the IL-6 mRNA levels.**
(DOCX)
