# Supplementary material for: Perilipin 2 (PLIN2)-Deficiency Does Not Increase Cholesterol-Induced Toxicity in Macrophages
Source: PLoS One. 2012 Mar 12;7(3):e33063. doi: 10.1371/journal.pone.0033063 (PMC3299742; doi:10.1371/journal.pone.0033063)
Supplement: Table S1 — Primer sequences. (DOCX) [file pone.0033063.s001.docx]

**Table S1.** Primer sequences.

| **Name Sequence** |
| --- |
| **PLIN2** Forward 5’-GATTCATTCACGTGGCCTCT- 3’  Reverse 5’-GGGAAGGAAAAACCTCACCT-3’ |
| **CHOP** Forward 5’-GGCATCACCTCCTGTCTGTC-3’  Reverse 5’-CTACCCTCAGTCCCCTCCTC-3’ |
| **XBP-1** Forward 5’-GAACCAGGAGTTAAGAACACG-3’  Reverse 5’-AGGCAACAGTGTCAGAGTCC-3’ |
| **IL-6** Forward 5’-TGACAATATGAATGTTGGGACA-3’  Reverse 5’-TTCCAAGAAACCATCTGGCTA-3’ |
| **TNF-α** Forward 5’-GTCCTGGAGGACCCAGTGT-3’  Reverse 5’-GGGAGCAGAGGTTCAGTGAT-3’ |
| **Cyclophilin A** Forward 5’-TGGTCTTTGGGAAGGTGAAA -3’  Reverse 5’-CACAGTCGGAAATGGTGATCT-3’ |
