# Supplementary material for: Perilipin 2 (PLIN2)-Deficiency Does Not Increase Cholesterol-Induced Toxicity in Macrophages
Source: PLoS One. 2012 Mar 12;7(3):e33063. doi: 10.1371/journal.pone.0033063 (PMC3299742; doi:10.1371/journal.pone.0033063)
Supplement: Table S4 — Three way ANOVA analysis of the IL-6 mRNA levels. (DOCX) [file pone.0033063.s004.docx]

**Table S4.** Three way ANOVA analysis of the IL-6 mRNA levels.

| **Independent Variable** | **Comparison** | **P value** | **Critical P Level** | **Significant?** |
| --- | --- | --- | --- | --- |
| **ADFP Genotype** | Wild-type *vs.* knockout | 0.649 | 0.050 | No |
| **FBS in media** | +FBS *vs.* -FBS | <0.00001 | 0.050 | Yes |
| **Treatment** | 7-KC *vs.* Untreated | <0.00001 | 0.005 | Yes |
|  | 7-KC *vs.* acLDL | <0.00001 | 0.003 | Yes |
|  | 7-KC *vs.* ACATi | <0.00001 | 0.004 | Yes |
|  | 7-KC *vs.* acLDL+ACATi | <0.00001 | 0.004 | Yes |
|  | 7-KC *vs.* EtOH | <0.00001 | 0.004 | Yes |
|  | acLDL+ACATi *vs.* Untreated | 0.0164 | 0.006 | No |
|  | acLDL+ACATi *vs.* acLDL | 0.631 | 0.017 | No |
|  | acLDL+ACATi *vs.* ACATi | 0.512 | 0.013 | No |
|  | acLDL+ACATi *vs.* EtOH | 0.757 | 0.050 | No |
|  | acLDL *vs.* Untreated | 0.00463 | 0.005 | Yes |
|  | acLDL *vs.* ACATi | 0.259 | 0.009 | No |
|  | acLDL *vs.* EtOH | 0.431 | 0.010 | No |
|  | ACATi *vs.* Untreated | 0.0738 | 0.007 | No |
|  | ACATi *vs.* EtOH | 0.729 | 0.025 | No |
|  | Untreated *vs.* EtOH | 0.0345 | 0.006 | No |
